# In Vitro Comparison between Metal Sleeve-Free and Metal Sleeve-Incorporated 3D-Printed Computer-Assisted Implant Surgical Guides

**DOI:** 10.3390/ma14030615

**Published:** 2021-01-29

**Authors:** Kyung Chul Oh, June-Sung Shim, Ji-Man Park

**Affiliations:** Department of Prosthodontics, Yonsei University College of Dentistry, Seoul 03722, Korea; kyungabc@yuhs.ac (K.C.O.); jfshim@yuhs.ac (J.-S.S.)

**Keywords:** computer-assisted implant surgical guide, metal sleeve-free implant surgical guide, metal sleeve-incorporated surgical guide, additive manufacturing, angular deviation, fully guided implant placement

## Abstract

The present study aims to compare the accuracy of metal sleeve-free 3D-printed computer-assisted implant surgical guides (MSF group) (*n* = 10) with metal sleeve-incorporated 3D-printed computer-assisted implant surgical guides (MSI group) (*n* = 10). Implants of diameter 4.0 mm and 5.0 mm were placed in the left second premolars and bilateral first molars, respectively, using a fully guided system. Closed-form sleeves were used in teeth on the left and open-form sleeves on the right. The weight differences of the surgical guides before and after implant placement, and angular deviations before and after implant placement were measured. Weight differences were compared with Student’s *t*-tests and angular deviations with Mann–Whitney tests. Cross-sectional views of the insert parts were observed with a scanning electron microscope. Preoperative and postoperative weight differences between the two groups were not statistically significant (*p* = 0.821). In terms of angular deviations, those along the mesiodistal direction for the left second premolars were significantly lower in the MSF group (*p* = 0.006). However, those along the mesiodistal direction for the bilateral molars and those along the buccolingual direction for all teeth were not significantly different (*p* > 0.05). 3D-printed implant surgical guides without metal sleeve inserts enable accurate implant placement without exhausting the guide holes, rendering them feasible for fully guided implant placement.

## 1. Introduction

Prosthetically driven implant placement has long been considered fundamental for the long-term success of dental implants [1,2]. However, it was not until the 1990s that this protocol was clinically implemented. The emergence of cone-beam computed tomography (CBCT) scanning and the development of dental implant planning software have significantly contributed to achieving this goal [3,4]. Currently, additive manufacturing or 3D printing is commonly used in dentistry, with its most extensive application being the fabrication of computer-assisted surgical stents or guides [5,6]. Compared to milling technology, it decreases the laboratory time and reduces material consumption [7].

Surgical stents help clinicians locate the initial entry point of implants and partially navigate the direction and angulation of the implants [8,9]. In contrast, surgical guides differ from surgical stents as they aim to accurately navigate the direction, depth, and angulation of implants [9,10]. Several comparative in vitro and in vivo studies evaluating the accuracy of surgical stents and guides have been performed. These studies reported that the mean angular deviation of dental implants placed with the aid of surgical stents and surgical guides ranged from 4.65° to 7.79° and 2.30° to 4.16°, respectively, indicating the superior accuracy of fully guided implant placement system [9,11,12,13].

Sleeves play an important role in achieving this purpose. These are equipped as prefabricated metal forms (i.e., metal sleeve) or their information is incorporated in the designing software of the surgical guide so that they can be manufactured simultaneously during additive manufacturing of surgical guides (i.e., metal sleeve-free) [14]. Studies that compared the accuracy of implant placement in relation to the pre-planned implant positions with the use of surgical guides commonly used surgical guides that required metal sleeve insertion; industrial 3D printers were used to fabricate the body of the surgical guides, and the metal sleeves were inserted [12,15,16,17,18,19,20,21,22]. As in-office 3D printers are becoming more popular and their accuracy is being reported as comparable to that of industrial printers, metal sleeveless surgical guides are becoming a major trend [23,24,25].

However, the diameter of the sleeves in metal sleeveless surgical guides may be prone to distortion and may cause variations in the inner diameter of the sleeves, depending on the build-angle condition during additive manufacturing [26]. In addition, debris may easily accumulate around the surgical sites [27]. In contrast, metal sleeves may be fabricated with a larger tolerance to allow the rotation of the guide drills, as these sleeves are more resistant to trimming [28]. It can be hypothesized that these characteristics may affect both the safety and accuracy of implant placement.

Hence, the present study aimed to compare the safety and accuracy of metal sleeve-free 3D-printed computer-assisted implant surgical guides with metal sleeve-incorporated 3D-printed computer-assisted implant surgical guides in a fully guided implant placement system. The loss of any material component was measured by the weight difference, and the accuracy of the implant placement was evaluated by measuring the angular deviation. The null hypothesis was that the weight difference of the surgical guides and the angular deviation of the implants before and after implant placement would differ between the two types of surgical guides.

## 2. Materials and Methods

### 2.1. Preparation of the Typodonts and Classification of the Groups

**Figure 1:**
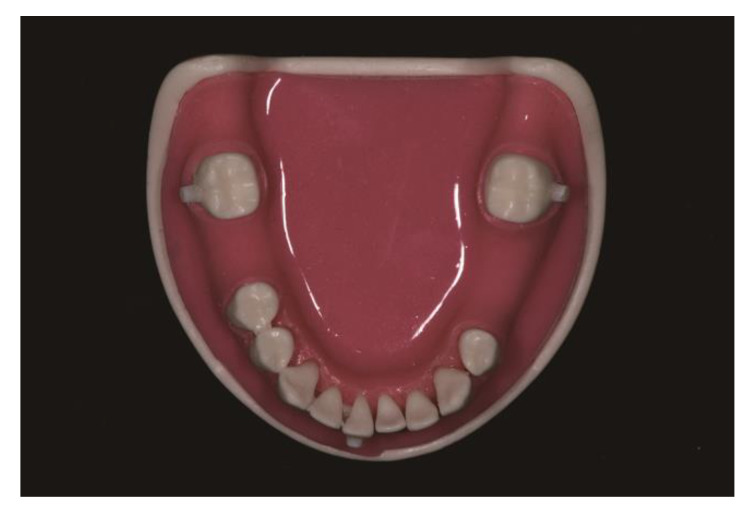
Mandibular typodont (CIBM01, Osstem, Busan, Korea) used in the present study. Radiopaque markers (Tetric N-Ceram, Ivoclar Vivadent, Schaan, Liechtenstein) were attached to the buccal surfaces of the bilateral second molars and the labial surface of the right central incisor.

A total of 20 mandibular typodonts (CIBM01, Osstem, Busan, Korea) with missing right first molars and left second premolars and first molars were used in this study. Radiopaque markers (Tetric N-Ceram, Ivoclar Vivadent, Schaan, Liechtenstein) were attached to the buccal surfaces of the bilateral second molars and the labial surface of the right central incisor (Figure 1). Ten typodonts received implant placement with metal sleeve-free 3D-printed computer-assisted implant surgical guides (*n* = 10, MSF group) for all implants, whereas the remaining ten received implant placement with metal sleeve-incorporated 3D-printed computer-assisted implant surgical guides (*n* = 10, MSI group) for all implants.

### 2.2. Design and Fabrication of the Computer-Assisted Implant Surgical Guides

Each typodont was scanned using a tabletop scanner (T500, Medit Co., Seoul, Korea) and a CBCT scanner (PHT-30LFO, Vatech, Seoul, Korea). The resultant standard tessellation language (STL) file and the Digital Imaging and Communications in Medicine files were imported into an implant planning software program (Implant Studio version 1.7.3.2, 3Shape A/S, Copenhagen, Denmark). The three radiopaque markers served as references to align the surface scan data obtained with the tabletop scanner and the CBCT scan data by a 3-point matching method.

Placement of a 4.0 mm diameter implant (TSIII, Osstem) was planned for the left second premolar, and that of 5.0 mm diameter implants (TSIII) was planned for the bilateral first molars. The guides were designed such that the implants could be placed under complete guidance using a fully guided surgical kit (OneGuide, Osstem). For the left second premolar and first molar, the sleeves were designed in a closed form, whereas for the right first molar, the sleeve was designed in an open form. For the MSI group, an additional step was included within the workflow of the software program to select the metal sleeves before finalizing the guide design.

**Figure 2:**
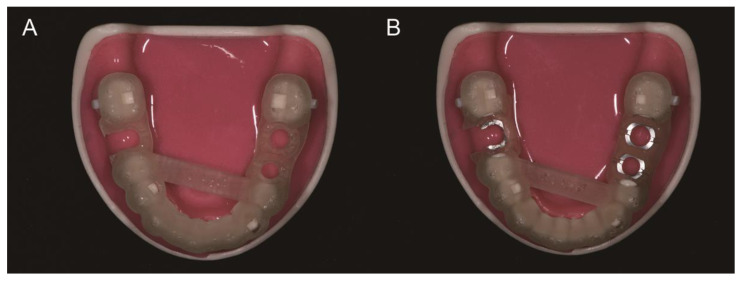
3D-printed computer-assisted implant surgical guides fabricated with a 3D printer (NextDent 5100, 3D Systems, Rock Hill, SC, USA) and a resin material (NextDent SG, 3D Systems). (**A**) Metal sleeve-free surgical guide (MSF group). (**B**) Metal sleeve-incorporated surgical guide (MSI group).

All surgical guides were fabricated one-by-one by a 3D printer (NextDent 5100, 3D Systems, Rock Hill, SC, USA) that uses a continuous digital light processing technology and a resin material (NextDent SG, 3D Systems). All guides were printed with a 30° build orientation angle and a slicing resolution along the *z*-axis of 100 µm. The guides were soaked in isopropyl alcohol for 10 min and cured in a post-curing machine for 30 min (LC-3DPrint Box, 3D Systems). Subsequently, the supports were removed, and the guides were polished. For the MSI group, the sleeves were attached to the guides by means of cyanoacrylate glue (Loctite, Düsseldorf, Germany) (Figure 2).

### 2.3. Measurement of the Weight Difference of the Surgical Guides before and after Implant Placement

Prior to implant placement, the weight of each surgical guide was measured using an electronic scale (OHAUS Explorer EX324G, OHAUS Corp., Parsippany, NJ, USA). Immediately after implant placement, the weight of the surgical guides was re-measured with the same electronic scale.

### 2.4. Surgical Procedures

Drilling procedures were performed according to the manufacturer’s guidelines by an experienced clinician. Two identical sets of drills were prepared for the drillings and applied separately for each group. After the surgical guide was placed on its corresponding typodont, tissue punch drills were applied to the surgical sites to remove the gingival portion of the typodont. Subsequently, initial drills were applied, and then a tapered drill designated for implants with the corresponding implant diameter and a length of 7 mm was applied. Two additional tapered drills with the same diameter were used to finalize the drilling procedures. The drills were not rotated prior to their insertion into the sleeves to avoid unintentionally damaging the surgical guide surface around the sleeves. The drillings were conducted in a to-and-fro motion, and all drilling procedures were conducted under profound saline irrigation, with the drilling speeds ranging from 800 to 1200 rpm. Finally, the implants were inserted with the surgical guides placed on the typdont. The insertion torques ranged from 30 to 50 N cm.

### 2.5. Measurement of the Angular Deviation of the Implants

**Figure 3:**
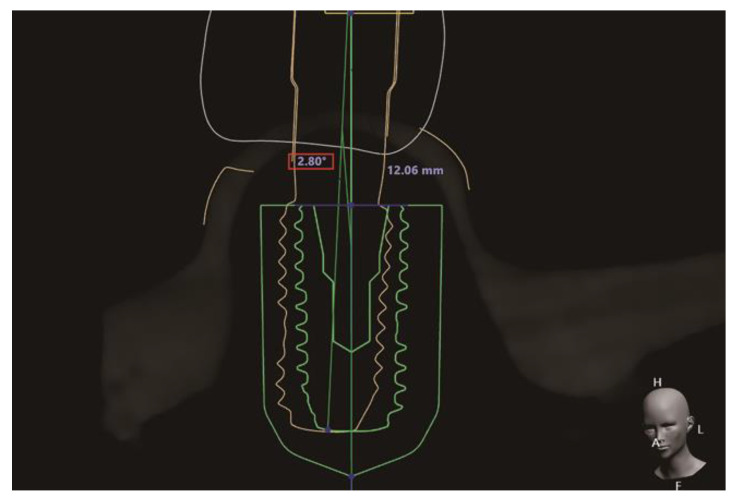
Representative image of the measurement method of the preoperative and postoperative angular deviation of the implant using the implant planning software program (Implant Studio version 1.7.3.2, 3Shape A/S, Copenhagen, Denmark). The green line indicates the contour of the implant in the preoperative stage, whereas the yellow line indicates the postoperative contour.

Scan bodies (TSSBM and TSBS, Osstem) were connected to the three implants on each typodont, scanned with a tabletop scanner (T500), and saved as an STL file. An extra 4.0 mm diameter implant and an extra 5.0 mm diameter implant were prepared and connected to the corresponding scan body individually and then scanned using the same tabletop scanner. The files were then saved in STL format. The implant-scan body assembly was superimposed over the typodont with scan bodies connected by means of the common part, using the best-fit alignment function in CAD software. This enabled the presentation of implant fixtures on the STL file of the typodont and was saved as another STL file. This STL file was re-imported into the same implant-planning software program [29]. The angulation between the preoperative and postoperative stages of the implant was measured from both the mesiodistal and buccolingual perspectives (Figure 3).

### 2.6. Scanning Electron Microscopic (SEM) Analysis

Cross-sectional surfaces of the metal sleeve-free insert parts and metal sleeve inserts were obtained before and after the drilling procedures. The surfaces were coated with platinum using a sputter coater (E-1010, Hitachi, Ltd., Tokyo, Japan) with a 100 nm thickness. The specimens were observed with a scanning electron microscope (Hitachi S-3000 N, Hitachi, Ltd.) at an accelerating voltage of 15 kV and under 18× and 80× magnifications.

### 2.7. Statistical Analysis

Student’s *t*-test was used to compare the mean weight difference between the two groups, and Mann–Whitney tests were used to compare the mean angular deviations. The data were analyzed using statistical software (IBM SPSS Statistics v23.0, IBM Corp, Armonk, NY, USA), with the level of significance set to α = 0.05.

## 3. Results

**Figure 4:**
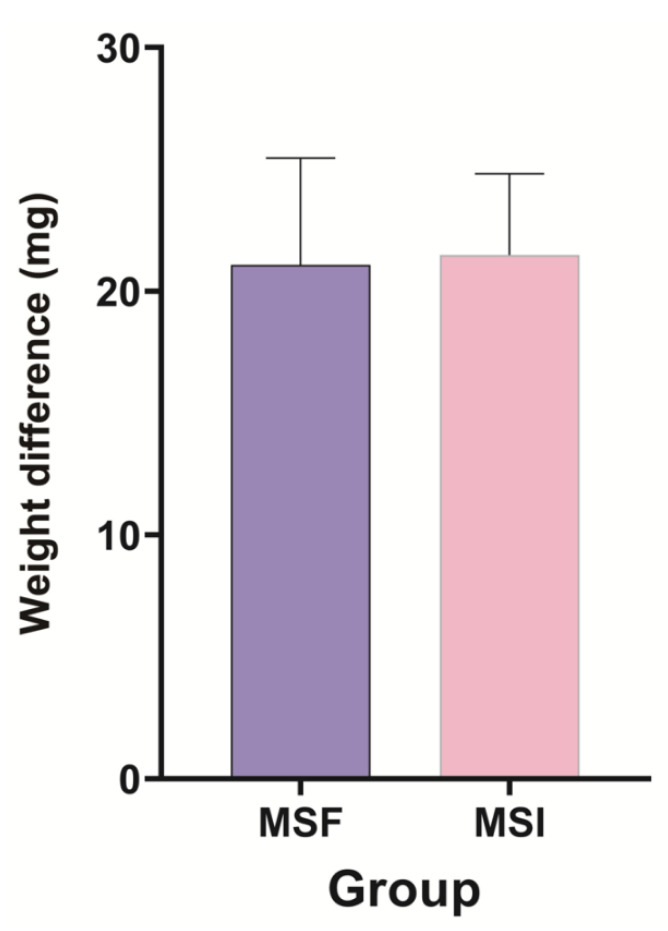
Preoperative and postoperative weight differences of the MSF and MSI groups. No statistically significant differences were observed. Data are expressed as mean ± standard deviations.

**Figure 5:**
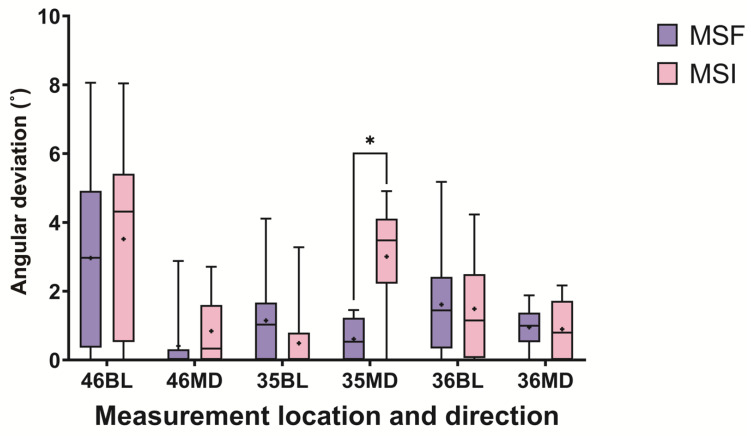
Preoperative and postoperative angular deviations of the MSF and MSI groups. The lines inside the boxes indicate median values, and the cross signs inside the boxes indicate mean values. The borders of the boxes indicate the 25th and 75th percentiles. The whiskers indicate the minimum and maximum values. The statistically significant difference is marked with an asterisk (*p* < 0.05). BL, buccolingual. MD, mesiodistal.

**Figure 6:**
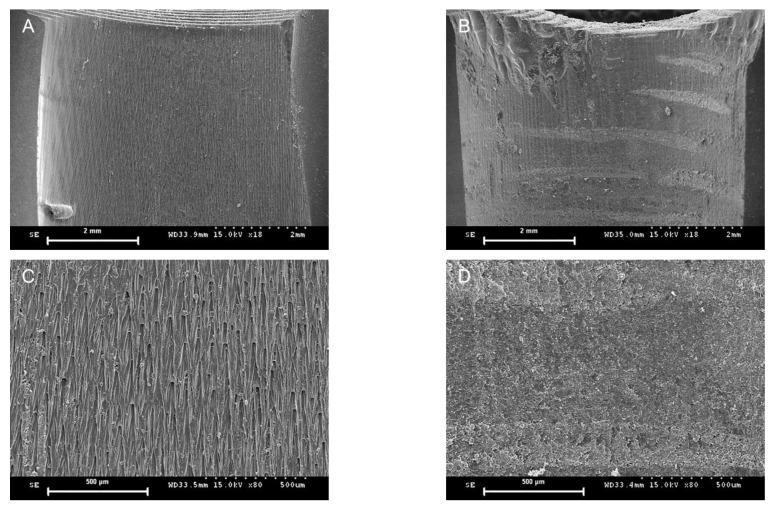
Representative scanning electron microscopic images. Cross-sectional view of a sleeve insert from a preoperative (**A**) and a postoperative (**B**) image from the MSF group in a lower magnification (18×). Cross-sectional view of a sleeve insert from a preoperative (**C**) and a postoperative (**D**) image from the MSF group at a higher magnification (80×).

**Figure 7:**
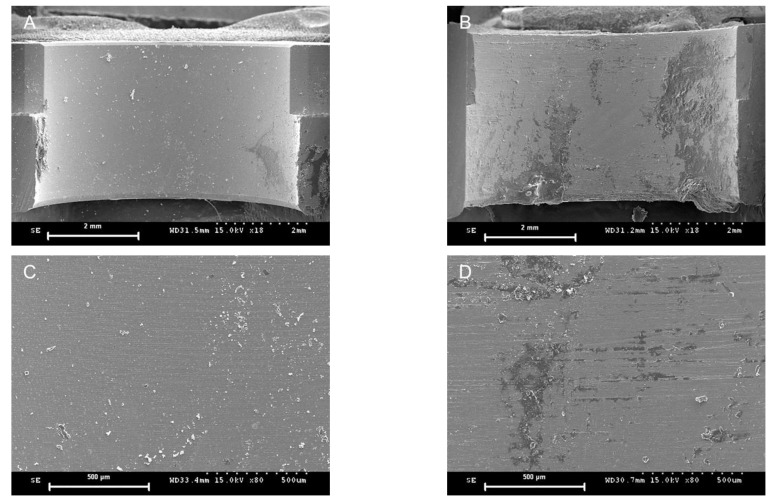
Representative scanning electron microscopic images. Cross-sectional view of a sleeve insert from a preoperative (**A**) and a postoperative (**B**) image from the MSI group at a lower magnification (18×). Cross-sectional view of a sleeve insert from a preoperative (**C**) and a postoperative (**D**) image from the MSI group at a higher magnification (80×).

All 20 implant surgical guides were successfully fabricated. The mean of the preoperative and postoperative weight differences of the surgical guides in the MSF and MSI groups were 21.09 mg and 21.49 mg, respectively. No statistically significant differences were observed between the two groups (*p* = 0.821) (Figure 4 and Table 1). Statistically significant differences were observed in terms of the angular deviations mesiodistally for the left second premolar area, with the MSF group exhibiting significantly smaller angular deviations (*p* = 0.006), while the differences were not significant buccolingually between the two groups (*p* = 0.125) (Figure 5 and Table 1). There were no statistically significant differences between the two groups either buccolingually or mesiodistally for the right first molar (*p* = 0.617 and *p* = 0.294, respectively) as well as either buccolingually or mesiodistally for the left first molar (*p* > 0.999 and *p* = 0.989, respectively). Pre-drilling, the SEM image of the MSF group appeared layered; however, after drilling, a squeezed area appeared (Figure 6). Pre-drilling, the SEM of the MSI group showed a smooth metal surface. Post-drilling, a significant black area appeared (Figure 7).

## 4. Discussion

3D printing is a key topic in various parts of industry as well as in the medical and dental fields. Nowadays, its application is not only limited to a macroscopic scale but has expanded to the microscopic cellular levels in which the technology can manufacture functional scaffolds for biomedical applications [30,31]. The biggest advantage of this technology is that it enables personalized fabrication of the object from a large variety of materials [32,33]. In the present study, a monomer based on acrylic esters and a digital light processing 3D printer were used to fabricate the surgical guides.

The null hypothesis that the weight difference between the two types of surgical guides before and after implant placement would differ was rejected, whereas the other null hypothesis that the preoperative and postoperative angular deviation of the implants would differ was accepted. The guide drills were prepared individually for the two groups in order to avoid unwanted damage to the surgical sites that may arise from wearing out of the drills. In this study, the guides of both groups were in-office 3D-printed, as this was considered adequate to achieve comparable accuracy to that of industrial 3D printers for clinical purposes.

The three implantation sites in each typodont were designed with different guide holes and implant diameter combinations to represent different clinical situations: an open sleeve with a wide-diameter implant for the right first molar area, closed sleeve with a wide-diameter implant for the left first molar area, and closed sleeve with a narrow-diameter implant for the left second premolar area. Open sleeves can be useful in the molar areas as they can be used with a smaller amount of mouth opening, making it possible to perform implant drilling in cases with an insufficient interarch space. The sleeves are usually C-shaped with the open part facing buccally; therefore, placing an implant with an open sleeve design surgical guide on a premolar was precluded.

Concerns about using surgical guides include the inner surface of the sleeves being trimmed during the drilling procedures and the components of the surgical guides being embedded in the surgical site during the drilling process. It would be best to assess the change on the inner surface of the sleeve. However, this change may be hardly detectable considering the small differences. Alternatively, evaluation of the weight difference pre- and postoperatively can be an indirect, practical method to evaluate this concern.

The weight was measured immediately before and after implant placement to exclude potential distortion of the 3D-printed resin materials. The weight results indicated that the metal sleeves and metal-free sleeves were vulnerable to trimming with a series of guide drills composed of metal components. This was confirmed by the SEM image analyses. In the MSI group, the preoperative SEM images showed a smooth metal surface, in contrast to the layered appearance of the MSF group. This layering occurs due to the principle of additive manufacturing technology in which an object is built-up layer-by-layer. However, both groups showed postoperative signs of depression, as identified through the images. In the MSF group, this appeared as a pressed, flat, and squeezed surface, as if the skin were pressed against a glass window. In the MSI group, the area that experienced friction due to contact with the guide drill was highly polished and appeared as a dark area. This suggests that the tolerance with the guide drill of the MSF group may be tighter than that of the MSI group.

Previous studies have used both preoperative and postoperative CBCT scan datasets for measuring the angular deviation [9,34,35]. However, a limitation exists in this protocol. CBCT scan datasets might differ owing to the different orientation of the patients/objects, and the quality of the postoperative CBCT scan datasets can be affected by the implant-induced metal artifacts. Hence, the superimposition of the two CBCT scan datasets may not be accurate. The method for angular deviation measurement proposed in the present study used a scan body that allowed the addition of two different scan datasets by functioning as a shared common part. A recent study reported comparable outcomes of the scan body and postoperative CBCT methodology when used to evaluate the difference between the position of the implant in the preoperative planning stage and the actual implant position [29]. This protocol may be particularly beneficial in clinical situations, as it does not require additional CBCT scans.

The angular deviation was evaluated because it is a parameter directly related to the attrition of the inner part of the sleeves. The buccolingual angular deviation of the implants placed at the right first molar area exhibited a higher value than the other implants, although the values were not statistically analyzed. This may be attributed to the characteristics of the open sleeve form design per se, which cannot completely guide the drill path. The statistically significant difference observed in the mesiodistal angular deviation of the left second premolar area may be attributed to the intrinsic tolerance of the metal sleeve per se, as well as to the errors during the attachment of the metal sleeves to the surgical guides, leading to incomplete adaptation of the metal sleeves. However, the difference of 2.40° between the two groups may be considered clinically acceptable. It can be assumed that the cross-section of the metal sleeve-free inserts may not conform to a complete circle but an oval form according to the build angle set for 3D printing. However, the results indicate that the build angle used in the present study was suitable for additive manufacturing of the guides. Moreover, in contrast to metal inserts that require additional costs and procedures for attachment, metal sleeve-free inserts are cost-effective. Therefore, metal sleeveless surgical guides may outweigh the metal sleeve-incorporated guides with the 3D printer used in the present study.

However, careful interpretation is mandatory, as the present study has a few limitations. First, the study was in vitro. The model was scanned with a tabletop scanner, the alveolar bone and soft tissue portions were not reproduced as in the clinical situation, and the study did not reflect patient movements or simulate the presence of saliva or blood. Second, only a single 3D printer and a fully guided implant surgery system were evaluated. Third, the angular deviation may differ according to the method of determination of the cross-sectional plane for the angular analysis. Further studies with different 3D printer systems and guided implant surgical kits, and subsequent clinical studies with a split-mouth study design, will be of great value to verify the findings of the present study. Moreover, comparative studies using computer-assisted implant surgical guides, navigation surgery systems, and robots will be crucial to evaluate the digital technology applicability to the field of implant surgery [36].

## 5. Conclusions

Within the limitations of the present study, the following conclusions can be drawn. First, on combining the two analyses, the performance of the MSF group was comparable to that of the MSI group, demonstrating the feasibility and validity of fabricating the surgical guides with an additive technology alone, that is, without attaching metal sleeves. Second, the sleeve inserts in the MSF and MSI groups exhibited noticeable changes after the drilling procedures with guide drills.

## Figures and Tables

**Table 1:** Weight difference of the surgical guides prior to and following implant placement and angular deviations prior to and following implant placement between the MSF and MSI groups. Data are expressed as mean ± standard deviations. BL, buccolingual. MD, mesiodistal.

Group		MSF	MSI
Weight difference (mg)		21.09 ± 4.38	21.49 ± 3.34
Angular deviation (°)	Measurement location and direction		
	46BL	2.97 ± 2.62	3.52 ± 2.76
	46MD	0.41 ± 0.95	0.84 ± 1.07
	35BL	1.15 ± 1.38	0.49 ± 1.04
	35MD *	0.61 ± 0.65	3.01 ± 1.68
	36BL	1.62 ± 1.65	1.49 ± 1.46
	36MD	0.96 ± 0.57	0.90 ± 0.96

An asterisk indicates the statistically significant difference (*p* < 0.05).

## Data Availability

Data available on request due to restrictions eg privacy or ethical.
